# Prion Peptide Uptake in Microglial Cells – The Effect of Naturally Occurring Autoantibodies against Prion Protein

**DOI:** 10.1371/journal.pone.0067743

**Published:** 2013-06-28

**Authors:** Yvonne Roettger, Inga Zerr, Richard Dodel, Jan-Philipp Bach

**Affiliations:** 1 Department of Neurology, Philipps-University Marburg, Marburg, Germany; 2 Department of Neurology and National Reference Center for Surveillance of Transmissible Spongiform Encephalopathies, Georg August University Göttingen, Göttingen, Germany; The Scripps Research Institute Scripps Florida, United States of America

## Abstract

In prion disease, a profound microglial activation that precedes neurodegeneration has been observed in the CNS. It is still not fully elucidated whether microglial activation has beneficial effects in terms of prion clearance or whether microglial cells have a mainly detrimental function through the release of pro-inflammatory cytokines. To date, no disease-modifying therapy exists. Several immunization attempts have been performed as one therapeutic approach. Recently, naturally occurring autoantibodies against the prion protein (nAbs-PrP) have been detected. These autoantibodies are able to break down fibrils of the most commonly used mutant prion variant PrP106-126 A117V and prevent PrP106-126 A117V-induced toxicity in primary neurons. In this study, we examined the phagocytosis of the prion peptide PrP106-126 A117V by primary microglial cells and the effect of nAbs-PrP on microglia. nAbs-PrP considerably enhanced the uptake of PrP106-126 A117V without inducing an inflammatory response in microglial cells. PrP106-126 A117V uptake was at least partially mediated through scavenger receptors. Phagocytosis of PrP106-126 A117V with nAbs-PrP was inhibited by wortmannin, a potent phosphatidylinositol 3-kinase inhibitor, indicating a separate uptake mechanism for nAbs-PrP mediated phagocytosis. These data suggest the possible mechanisms of action of nAbs-PrP in prion disease.

## Introduction

Prion diseases are a group of transmissible neurodegenerative diseases characterized by progressive neuronal cell death, astrogliosis and microglial activation, leading to a spongiform degeneration of the central nervous system (CNS). The hallmark of the disease is the conversion of the physiological cellular prion protein (PrP^C^) into its isoform called scrapie prion protein (PrP^Sc^). This conversion is followed by further oligomerization and fibrillation, which has a pathological effect on cells. PrP^Sc^ is characterized by high β-sheet content, protease resistance and a potential to accumulate into aggregates [1], [2].

Recently, autoantibodies against the prion protein (nAbs-PrP) have been detected [3]. They are able to block the fibrillation into aggregates of prion peptides *in vitro* and can further reduce the toxicity of the peptides on cultured primary neurons. Naturally occurring autoantibodies (nAbs) are part of the innate immune system and make up 2/3 of the total IgG in humans [4]. nAbs have also been detected against other aggregating proteins, including β-amyloid (Aβ), tau and α-synuclein, and their role in neurodegenerative diseases is a major topic of current research [3], [5], [6], [7].

Prion-induced toxicity to neuronal cells depends on PrP^C^ expression, as cells from PrP^0/0^ mice are not susceptible to prion exposure [8]. The toxicity of prion peptides to neuronal cells further requires the presence of microglial cells [9], [10]. The release of destructive oxidants by microglial cells is involved in this mechanism [8]. Microglial cells play an important role in neurodegenerative diseases such as Alzheimer’s disease. However, it is still controversial whether they play a protective role by secreting neurotrophic and anti-inflammatory molecules and support the clearance of accumulated proteins or whether they contribute to disease progression by releasing several cytotoxic substances, such as nitric oxide (NO) and pro-inflammatory cytokines [11]. In prion disease, microglial activation precedes neuronal cell death, indicating a potential detrimental role of microglia [12]. On the other hand, microglia internalize PrP^Sc^ and prion peptides, suggesting a clearance activity of microglial cells in prion disease [13].

Therefore, we aimed to analyze whether primary microglial cells phagocytose PrP106-126 A117V and whether this uptake can be influenced by nAbs-PrP. PrP106-126 A117V is a synthetic peptide carrying residues 106-126 of human prion protein with an A117V mutation, which is linked to Gerstmann-Straeussler-Scheinker-syndrome. PrP106-126 A117V exhibits some of the physiochemical and pathogenic properties of PrP^Sc^, including the formation of fibrils and the ability to induce apoptosis in neuronal cells [14], [15]. Compared to PrP106-126, it forms fibrils even faster *in vitro* [16], and both peptides are widely used to mimic the effect of PrP^Sc^ *in vitro*.

## Materials and Methods

### Ethics Statement

All animal procedures were conducted according to current German law for animal welfare and were announced to the Regierungspräsidium Giessen (district president). Approval by an institutional animal welfare committee as such is not required for this type of experiment. According to current German law (TierSchG, §6 Abs. 1, Nr. 4), it is sufficient to announce these experiments to the district president (http://www.rp-giessen.hessen.de/irj/RPGIE_Internet?cid=efcda20a6301329333db4f7e186a14d1). All animal procedures were approved by the office of the district president and the Institutional Animal Care and Use Committee of the University of Marburg.

### Primary Cell Culture

Primary mouse cell cultures were prepared from embryonic day 13.5 (E13.5) Swiss Webster mice. Pregnant mice were purchased from Janvier at the stage when the embryo was at day 12.5 (*Janvier*, Le Genest-saint-Isle, France) and housed overnight in our animal-care facility until sacrifice. Mice were housed on a 12-hour light-dark schedule (lights on 07∶00–19∶00). They had free access to tap water, were fed *ad libitum* and were kept under standard conditions. The oldest embryo used during the latest period of this study was 13.5 days. After pregnant dams were sacrificed by cervical dislocation by experienced researchers, embryos were removed from the uterus. Embryonic death was confirmed by dislocating their heads before preparation of the brain.

Mesencephalons were used for the preparation of microglial cell cultures to achieve highest yield of microglial cells [17]. Briefly, mesencephalons from embryonic brains, were collected in 2 ml Leibovitz L-15 medium (PAA Laboratories, Pasching, Austria) and homogenized by gently pipetting up and down 30 times. After the addition of 5 ml Leibovitz L-15 medium, the cell solution was left for 10 minutes to remove debris and 5 ml of the supernatant was transferred into a new tube. After centrifugation at 300 g for 5 min, the supernatant was discarded and the remaining pellet was resuspended in 1 ml Dulbeccòs modified Eaglès medium (DMEM with L-glutamine; Lonza, Basel, Switzerland) supplemented with 10% fetal calf serum (FCS) (PAA Laboratories), 100 U/ml penicillin and 100 µg/ml streptomycin (Lonza). Cells were cultured in polyethylenimine (PEI)-coated 6-well plates. To further improve the yield of microglial cells, the cell media was supplemented with 10 ng/ml GM-CSF (Roche, Basel, Switzerland) as previously described by Re et al. and Esen et al. [18], [19]. Instead of the trypsinization protocol and the replating of microglial cells described by Saura et al., we replated the cells without a preceding trypsinization step. Then we followed the protocol by Saura et al. and cells were cultured for 14 days until experimental use [20]. Cells were replated onto PEI-coated 24-well or 48-well plates in a density of 1–2×10^5^ cells per ml. Cells obtained by this protocol were mainly microglia (>95%) as quantified by CD11b staining using FACS analysis before experimental use of the cells.

For neuronal cells, embryonic cortices were isolated and prepared as described above. After centrifugation, cell pellets were resuspended in Neurobasal-A Medium (Invitrogen, Grand Island, NY, USA) supplemented with B27 (GIBCO, Basel, Switzerland), 100 U/ml penicillin, 100 µg/ml streptomycin and L-glutamine and plated in PEI-coated plates. Cells were used for experiments on day 7 *in vitro*.

### Peptides

Peptides were purchased by PSL - Peptide Specialty Laboratories (PSL, Heidelberg, Germany). These peptides included PrP106-126 A117V with the sequence KTNMKHMAGAVAAGAVVGGLG as well as PrP106-126 A117V with an N-terminal fluorescein isothiocyanate (FITC).

### Purification of nAbs-PrP and ft-PrP

Antibody preparations were isolated from intravenous immunoglobulin (IVIg) as previously described [3]. Briefly, disposable chromatography columns were packed with UltraLink Iodoacetyl Gel (Thermo Scientific, Rockford, IL, USA). PrP106-126 A117V was conjugated to the matrix according to manufacturer`s instructions. IVIg (1∶1 in PBS) was loaded on the columns overnight at 4°C. Unbound fractions (IVIg depleted of nAbs-PrP, termed flow-through (ft-PrP)) were passed through the columns and collected. After several washes, bound IgG fractions were released and collected by passing elution buffer (50 mM glycine at pH 2.5) through the column. As was shown by Wei et al. (2012), nAbs-PrP bind to the extreme N-terminus of PrP106-126 [3]. The binding requires, at a minimum, residues KTNMK (PrP106-110), with both lysine residues being critical for high-affinity antibody binding. nAbs-PrP bound specifically to PrP106-126 A117V, as was shown by immunoprecipitating PrP106-126 A117V from homogenates of brains from transgenic mice expressing the human sequences encompassing residues 106–126 [3].

### Fibril Formation Assay

The formation of fibrils was quantified by the thioflavin T (ThT) binding assay [21]. Peptides were dissolved in PBS with or without antibodies at final concentrations of 150 µM peptide and 2.5 µM antibody. After incubation for 48 hours at 37°C, samples were added to 80 µl glycine buffer (50 µM, pH 9.2) and 10 µl ThT (4 µM working concentration). Fluorescence was measured using a Tecan Infinite M200 reader with excitation at 450 nm and emission at 485 nm.

### Cell Viability Assays

Peptides were prepared as described above. Microglial cells were treated with a final peptide concentration of 10 µM for 24 hours in DMEM without FCS. Viability was measured either using the 3-(4,5-dimethylthiazol-2-yl)-2,5-diphenyltetrazolium bromide (MTT) reduction assay and/or by staining with fluorescein diacetate/propidium iodide (FDA/PI). For the MTT assay, cells were incubated for 1 hour at 37°C with serum-free medium containing 0.5 mg/ml MTT. Cells were permeabilized with dimethyl sulfoxide (DMSO) (AppliChem, Darmstadt, Germany) for another 30 minutes, and absorbance was measured at 570 nm. For FDA/PI staining, cells were incubated with FDA and PI at final concentrations of 0.15 mg/ml and 0.02 mg/ml, respectively. For the treatment of neuronal cells with the supernatant of microglial cells, medium was removed, and the conditioned medium from treated microglial cells was added. The MTT assay was conducted after 24 hours. All measurements were performed at least in duplicate.

### Quantification of IL-6, TNF-α and NO Release

Primary microglial cells were stimulated with peptide preparations (10 µM) and LPS (1 µg/ml; extracted from *Escherichia coli,* Sigma). After 24 hours, the supernatant was collected, and IL-6 and TNF-α were quantified using the Duoset ELISA system (R&D, Minneapolis, MN, USA). The production of NO was measured by using the Griess reagent (1 mM sulfanilamide, 1 mM naphthalenediamine dihydrochloride, 100 mM HCL), which detects a degradation product, nitrite (NO_2_^−^). Absorbance was measured at 450 and 540 nm for the ELISA and Griess assay, respectively.

### Western Blot

Cell lysates were prepared according to the manufacturer’s protocol using M-Per solution (Thermo Scientific, Rockford, IL, USA) supplemented with protease inhibitor. Total cell protein was loaded onto 4–12% NuPage Bis-Tris gel (Invitrogen, Carlsbad, CA, USA) and electroblotted onto nitrocellulose membranes. Following blocking with 1× Roti-block (Carl Roth, Karlsruhe, Germany), membranes were stained with POD-conjugated anti-human IgG (Thermo Scientific, Rockford, IL, USA) (1/25000) or anti-α-tubulin antibody (Sigma, St. Louis, MO, USA) (1/5000). Binding was visualized by incubating membranes with Super Signal West Dura Extended Duration working solution (Thermo Scientific, Rockford, IL, USA) followed by exposure to an autoradiographic film (CL-Xposure Film, Thermo Scientific, Rockford, IL, USA).

### PrP106-126 A117V Uptake Assay

Fibrillation was carried out as described above using 150 µM FITC-labeled PrP106-126 A117V with or without 2.5 µM antibodies (nAbs-PrP or ft-PrP). Primary microglial cells were treated with PrP106-126 A117V-FITC for 3 hours at 37°C in DMEM without FCS. For flow-cytometric analysis, cells were harvested after several washes with ice-cold PBS. Cells were washed with fluorescence-activated cell sorting (FACS) buffer (PBS with 0.1% FCS) and probed with APC-conjugated CD11b antibody (eBioscience, San Diego, CA, USA) for 20 minutes at 4°C and protected from the light. After washing cells with FACS buffer, cells were stained with HOECHST 33258 (Sigma, St. Louis, MO, USA) to detect dead cells. Measurements were carried out with an LSR II flow cytometer (Becton Dickinson, Franklin Lakes, NJ, USA), and the analysis was performed using FlowJo software (Tree Star Inc., Ashland, OR, USA). Only CD11b-positive and Hoechst-negative cells were used for the evaluation of PrP106-126 A117V-FITC uptake. PrP106-126 A117V uptake was measured by the mean fluorescence intensity of the cells. The uptake of antibodies was determined by Western blot.

### Statistical Analysis

All results are presented as the mean ± SD. To assess statistical significance, we used Student’s *t* test. The following definitions were used: *p*<0.05 (*), *p*<0.01 (**) and *p*<0.001 (***).

## Results

### nAbs-PrP Block Fibril Formation of PrP106-126 A117V

**Figure 1:**
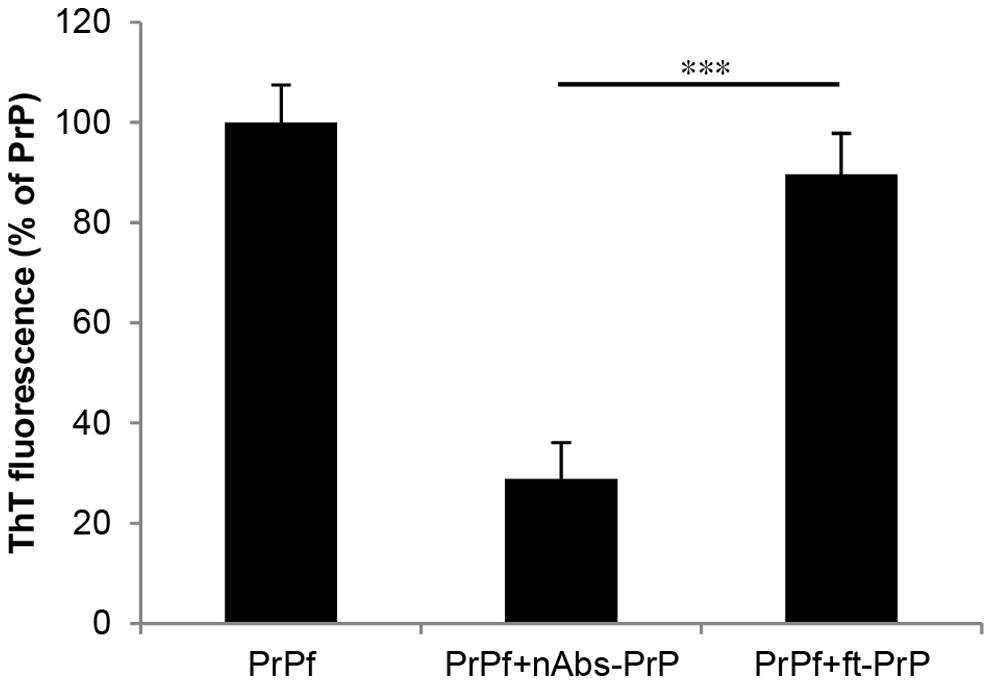
nAbs-PrP block fibril formation of PrP106-126 A117V. PrP106-126 A117V peptide (150 µM) was incubated with or without nAbs-PrP and ft-PrP at a ratio of 1∶60 at 37°C for 48 hours. The ThT Assay was performed to measure fibril formation. Fibril formation of PrP106-126 A117V was referred to as 100%. Experiments were performed at least three times independently.

All experiments were carried out using PrP106-126 A117V in its fibrillated form. For this purpose, the peptide was incubated either alone or with nAbs-PrP or ft-PrP for 48 hours at 37°C. Fibrillation was reduced by 70% when the peptide was incubated with nAbs-PrP, whereas co-incubation of the peptide with unspecific IgG (ft-PrP) did not reduce the fibril formation (Fig. 1). The monoclonal anti-PrP antibody 3F4 also reduced PrP106-126 A117V fibril formation by 60% (data not shown).

### Uptake of PrP106-126 A117V, nAbs-PrP and ft-PrP in Primary Microglial Cells

**Figure 2:**
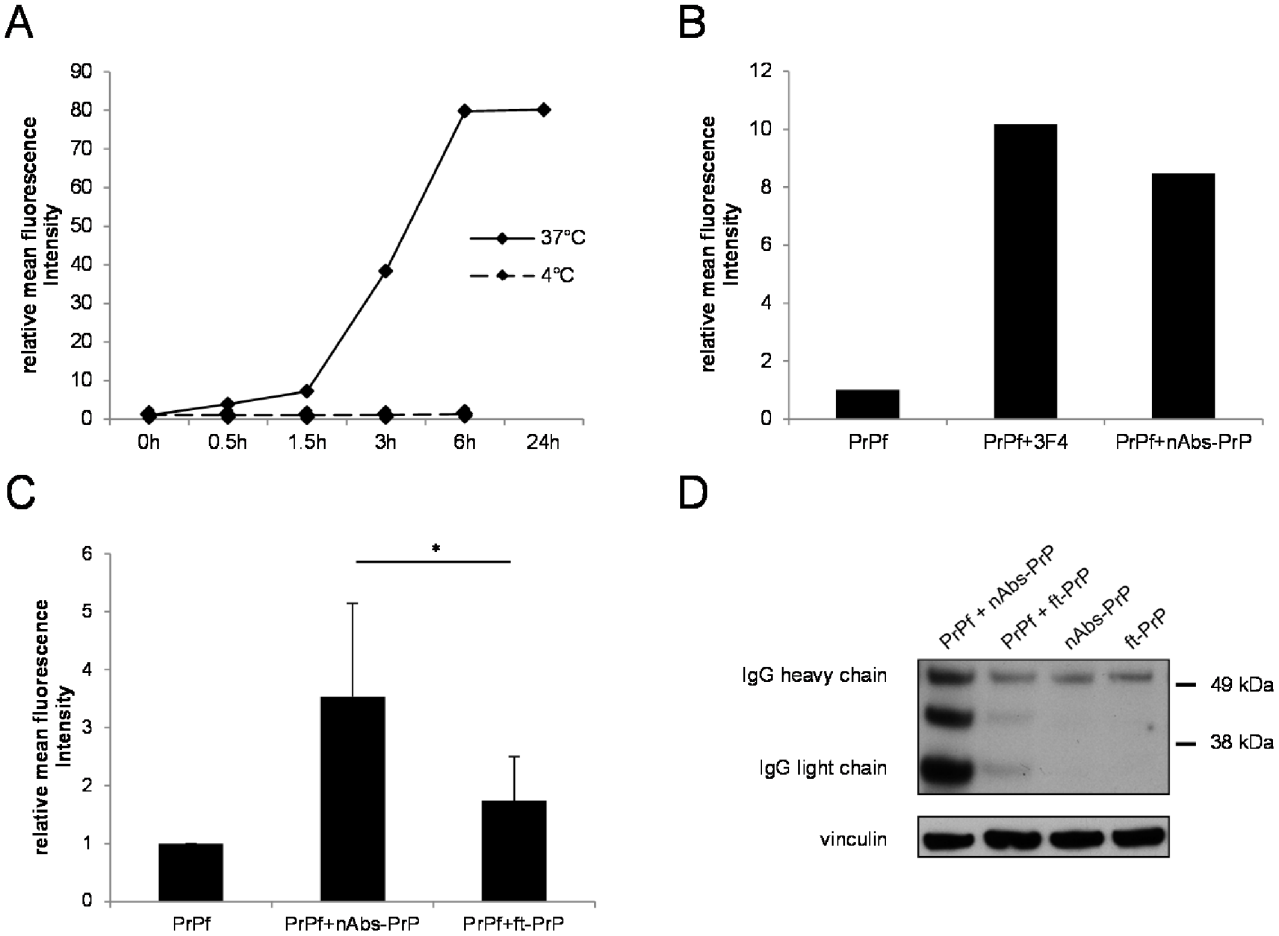
Uptake of prion fibrils, nAbs-PrP and ft-PrP in primary microglial cells. The uptake of PrP106-126 A117V fibrils (10 µM) was measured after 0, 0.5, 1.5, 3, 6 and 24 hour treatment by flow-cytometric analysis. Control experiments were conducted at 4°C to verify that this process was energy-dependent and not due to unspecific binding to the cells (A). To measure the antibody-mediated uptake of PrP106-126 A117V, cells were treated with preparations from the co-incubation of prion peptides with monoclonal antibody 3F4 or nAbs-PrP (B) for 3 hours. Values are normalized to untreated cells, and one representative experiment out of three is shown (A, B). (C) Uptake was measured following incubation of the cells with PrP106-126 A117V, with or without nAbs-PrP or ft-PrP. Values are normalized to PrP106-126 A117V fibril-treated cells, and data from three independent experiments are shown (C). Western blot analysis of antibody uptake in microglial cells was performed after treatment of the cells for 3 hours with nAbs-PrP, ft-PrP or the co-administration of PrP106-126 A117V and nAbs-PrP or ft-PrP (D). Membranes were probed with peroxidase-conjugated anti-human IgG to detect antibody uptake. α-Vinculin was used as a loading control. One representative experiment out of three is shown.

Prion peptides are taken up by neurons, microglia and astrocytes [13]. We found that microglial cells phagocytosed FITC-labeled PrP106-126 A117V fibrils in a time-dependent manner, with a maximum uptake after 6 hours when normalized to untreated cells (Fig. 2A). This effect was energy-dependent and was not due to unspecific binding of fibrils to the cells, as we verified by control experiments carried out at 4°C (Fig. 2A).

Because we demonstrated that nAbs-PrP, as well as the monoclonal anti-PrP antibody 3F4, prevented PrP106-126 A117V fibril formation, we next investigated the effect of nAbs-PrP on the phagocytic ability of microglia. PrP106-126 A117V fibrillation was carried out in the presence or absence of nAbs-PrP or 3F4, and cells were treated with those peptide preparations for 3 hours. To be able to measure either a drop or a rise in the uptake of PrP106-126 A117V when co-incubated with nAbs-PrP, we chose this intermediate uptake level for further experiments (compared with Fig. 2A). After cells were treated with co-incubations of labeled prion peptide and the monoclonal anti-PrP-antibody 3F4 or nAbs-PrP, a 10-fold increase in prion peptide uptake was observed (Fig. 2B). To rule out a non-specific antibody effect, we repeated the same experiment with ft-PrP. ft-PrP did not significantly increase the FITC-PrP106-126 A117V uptake of microglia compared to nAbs-PrP (Fig. 2C). To investigate whether primary microglial cells phagocytosed nAbs-PrP and ft-PrP, we performed Western blot analysis with microglial cell lysates after 3 hour treatment (Fig. 2D). Cells were either treated with antibodies alone (nAbs-PrP or ft-PrP) or co-incubated with a combination of PrP106-126 A117V and antibody (PrP106-126 A117V with nAbs-PrP or ft-PrP). Microglial cells phagocytosed nAbs-PrP to a slightly greater extent than ft-PrP. The co-administration of PrP106-126 A117V and nAbs-PrP led to a strong increase in nAbs-PrP uptake, whereas the co-administration of PrP106-126 A117V with ft-PrP led only to a slight increase of ft-PrP uptake.

### The Effect of PrP106-126 A117V on Viability and Activation of Microglial Cells

**Figure 3:**
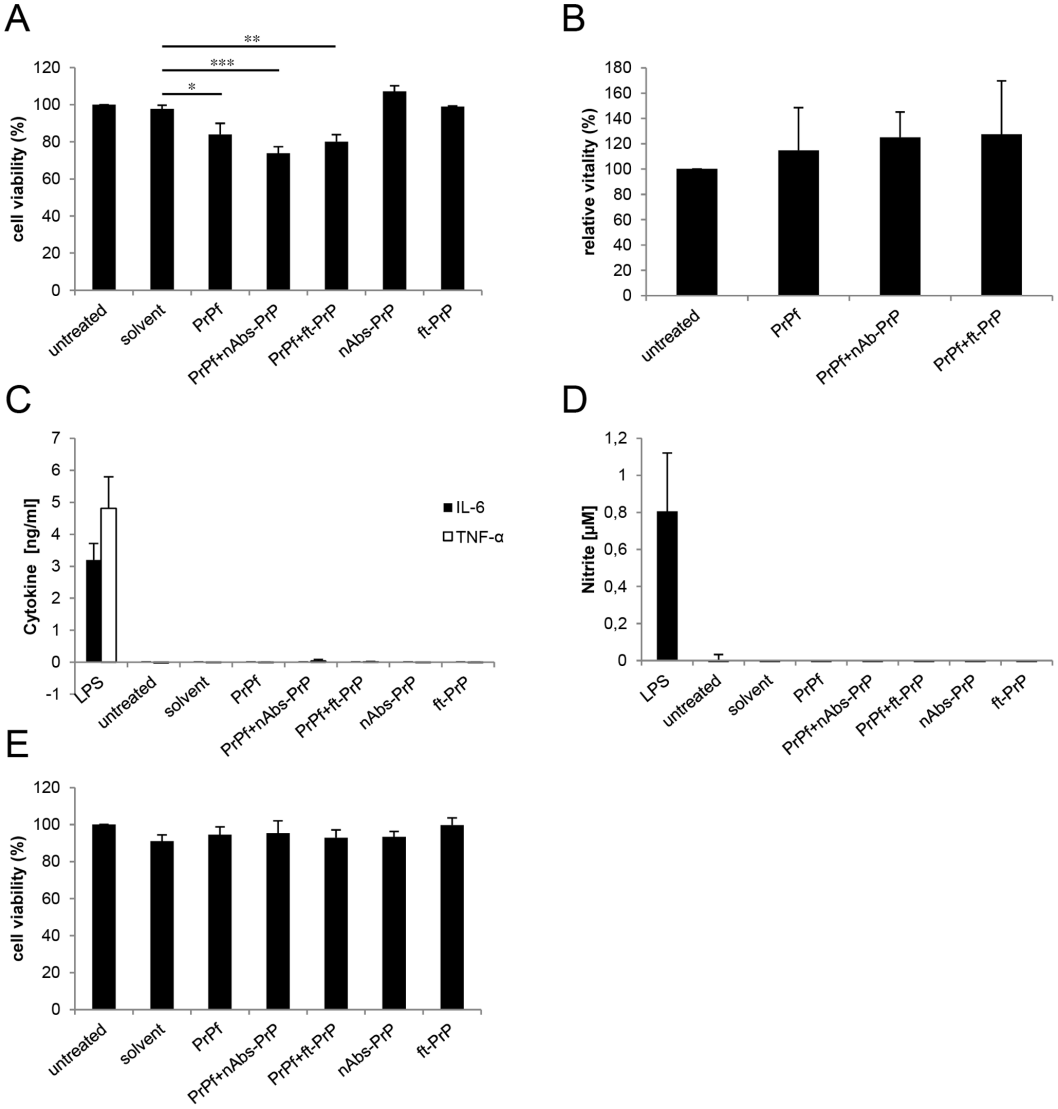
Effect of PrP106-126 A117V on microglia. Following treatment for 24 hours with 10 µM PrP106-126 A117V with or without nAbs-PrP or ft-PrP, the MTT assay was performed to measure the mitochondrial activity of microglial cells. Values are normalized to untreated cells (A). The vitality of treated cells was verified by staining microglia with fluorescein diacetate/propidium iodide, and values are normalized to untreated cells (B). Supernatants of the cells were subjected to cytokine ELISA (C) and Griess assay (D) with LPS (1 µg/ml) as the positive control. (E) Conditioned supernatant of microglial cells was administered to primary neurons for 24 hours, and the MTT assay was performed. Values are normalized to untreated cells. All experiments were performed at least three times independently.

To investigate the effect of prion peptide uptake on microglial cells, we examined the viability of treated cells by applying two different methods. To gain first insights into the reaction of microglial cells following a treatment with PrP106-126 A117V alone, with co-incubation of PrP106-126 A117V with antibodies (nAbs-PrP or ft-PrP) or with antibodies alone (nAbs-PrP or ft-PrP), we performed an MTT assay to measure mitochondrial activity (Fig. 3A). We examined a 15–25% reduction of the signals assessed by MTT assay following a treatment with PrP106-126 A117V alone or with a combination of PrP106-126 A117V and antibodies (nAbs-PrP or ft-PrP) when compared with control cells. Cells treated with nAbs-PrP or ft-PrP alone did not exhibit any differences in the signal intensity assessed by MTT assay compared to untreated cells. Amyloidogenic peptides have been shown to bear the ability to enhance the exocytosis of the reduced tetrazolium dye in cells [22]. Therefore, the use of MTT assay in combination with amyloidogenic peptides has limitations. To investigate whether the reduction in signal intensity examined by MTT assay following the treatment with PrP106-126 A117V preparations refer to a reduction of vitality of microglial cells, we additionally performed FDA/PI staining. No reduction in vitality was observed when counting living cells after the treatment with PrP106-126 A117V alone or with a combination of PrP106-126 A117V and nAbs-PrP or ft-PrP. Instead, the cells seemed to proliferate (Fig. 3B).

To investigate whether the treatment of microglial cells resulted in cytokine or NO release, concentrations of interleukin-6 (IL-6), tumor necrosis factor α (TNF-α) and NO were examined in the supernatant of microglial cells (Fig. 3C, D). No cytokine or NO release was observed (1 µg/ml LPS served as a positive control). To examine whether microglial cells exhibited detrimental function toward primary neuronal cells, we exposed neurons to conditioned microglial supernatant. No change in viability was observed (Fig. 3E).

### Inhibition of PrP106-126 A117V Uptake by Specific Blocker

To investigate the uptake mechanisms of microglia for PrP106-126 A117V, different uptake blockers were employed. Specific blockers were administered to the cells for 30 minutes (10 µM cytochalasin D, 500 µg/ml fucoidan) or 60 minutes (10 µM wortmannin) prior to the treatment with PrP106-126 A117V.

**Figure 4:**
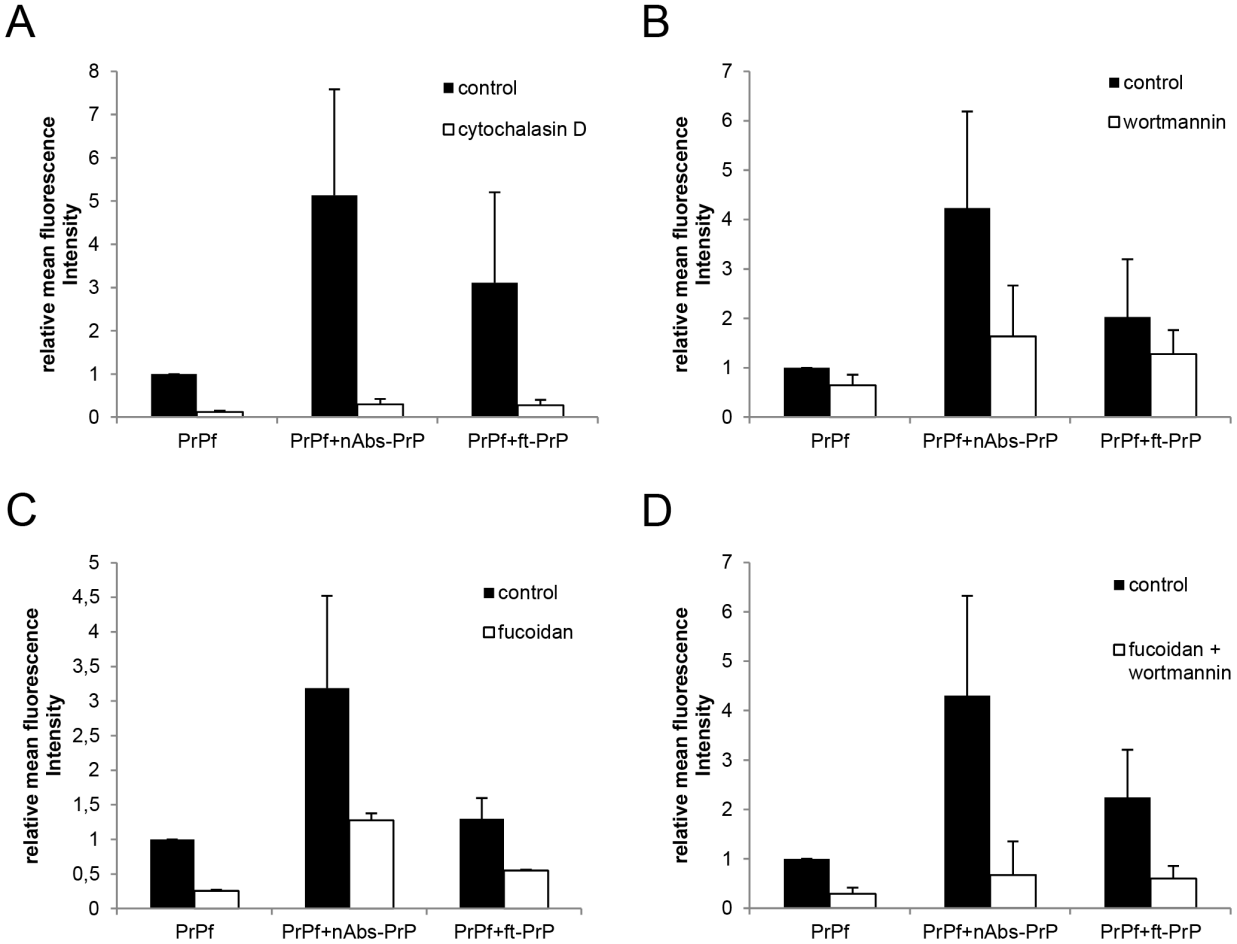
Inhibition of PrP106-126 A117V uptake by specific blockers. Phagocytosis assay was performed following pre-treatment of microglial cells with (A) cytochalasin D (10 µM, 30 minutes), (B) fucoidan (500 µg/ml, 30 minutes), (C) wortmannin (10 µM, 60 minutes) and (D) co-incubation with fucoidan and wortmannin. All experiments were performed at least three times independently, and values are normalized to PrP106-126 A117V fibril uptake.

Cytochalasin D interferes with microfilament function and inhibits the phagocytic activity of cells by depolymerizing actin [23], [24]. Pre-treatment of microglial cells with cytochalasin D (10 µM) resulted in an almost complete inhibition of uptake of all three PrP106-126 A117V preparations (PrP106-126 A117V alone or with nAbs-PrP or ft-PrP) (Fig. 4A).

Wortmannin is a potent inhibitor of phosphatidylinositol 3-kinase (PI3K) [25] and inhibits actin-dependent endocytosis, fluid-phase pinocytosis and phagocytosis [26]. Pre-treatment with wortmannin moderately reduced phagocytosis to 60% with regard to treatment with either PrP106-126 A117V fibrils or PrP106-126 A117V co-incubated with ft-PrP. It markedly reduced the uptake of PrP106-126 A117V co-incubated with nAbs-PrP (down to 38%) (Fig. 4B).

Fucoidan is an effective inhibitor of scavenger receptors A and B, which have been previously shown to mediate the uptake of Aβ in its fibrillar state [27], [28]. There was a marked inhibitory effect of fucoidan on the uptake of PrP106-126 A117V fibrils (25% of control), whereas uptake of PrP106-126 A117V co-incubated with nAbs-PrP was only reduced to 40% of the control level (Fig. 4C). Co-incubation with fucoidan and wortmannin did not further reduce uptake of PrP106-126 A117V fibrils alone (30%) but greatly reduced uptake of PrP106-126 A117V co-incubated with nAbs-PrP or ft-PrP (16% or 27%, respectively) (Fig. 4D).

### Wortmannin Inhibits the Uptake of nAbs-PrP and ft-PrP

**Figure 5:**
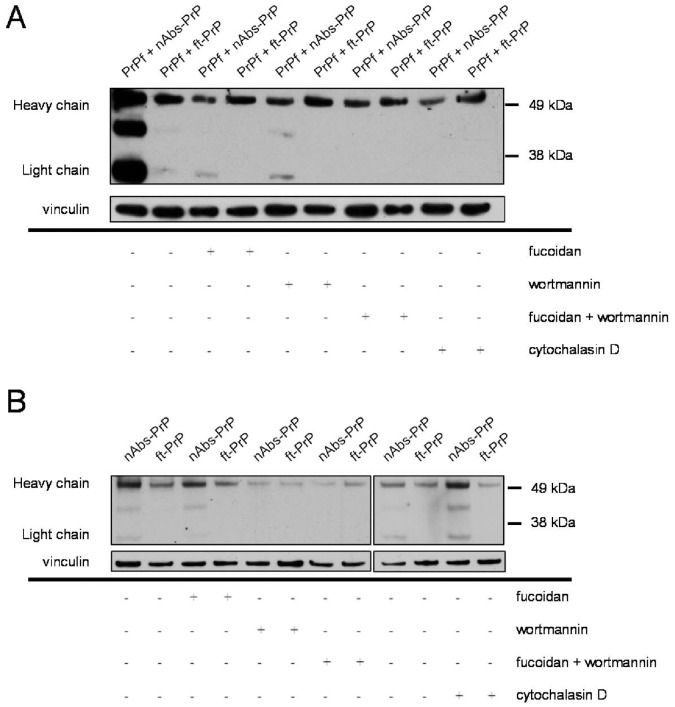
Inhibition of uptake of nAbs-PrP and ft-PrP by specific blockers. Western blot analysis of antibody uptake in microglial cells was performed following pre-treatment with cytochalasin D, fucoidan or wortmannin or the co-administration of fucoidan and wortmannin. Cells were co-incubated with PrP106-126 A117V and nAbs-PrP or ft-PrP (A) or with nAbs-PrP or ft-PrP alone (B). One representative experiment out of three is shown.

In addition to investigating the effects of specific inhibitors on the uptake of PrP106-126 A117V, we further examined the uptake of nAbs-PrP and ft-PrP. The results presented above revealed that the impact of the different phagocytosis blockers on the uptake of PrP106-126 A117V varied depending on whether it was co-incubated with nAbs-PrP or with ft-PrP (i.e., wortmannin blocked the uptake of PrP106-126 A117V co-incubated with nAbs-PrP to a greater extent than PrP106-126 A117V co-incubated with ft-PrP). This experiment showed a general inhibitory effect of all three blockers on the uptake of nAbs-PrP but not on the uptake of ft-PrP when either one was co-administered with PrP106-126 A117V (Fig. 5A). When nAbs-PrP or ft-PrP was administered without PrP106-126 A117V, fucoidan and cytochalasin D had no inhibitory effect on the uptake of the antibodies, whereas wortmannin greatly reduced the uptake of nAbs-PrP or ft-PrP (Fig. 5B).

## Discussion

In prion disease, a profound activation of microglial cells in regions with vacuolation, plaque formation and neuronal damage exists [29]. The exact role of microglia, however, is still not fully elucidated. In cell culture conditions, microglial cells aggregate around fibrillar PrP106-126 [30]. Continuous PrP106-126 exposure at high concentrations (e.g., 80 µM) induces cytokine production and the release of NO by microglia *in vitro* [9], [12]. Furthermore, PrP106-126 neurotoxicity in cell culture is induced by and dependent on the presence of microglial cells [8]. These results indicate that microglial cells have the potential to induce neuronal cell death via an inflammatory response. However, microglial cells have been considered to play a key role in prion clearance [31]. Falsing et al. (2008) observed a 15-fold increase in prion titers on organotypic cerebellar slices following ablation of microglia [32]. Kranich et al. (2010) considered the secreted ligand milk fat globule epidermal growth factor 8 (Mfge8) to be part of this potential clearance function of microglial cells [33]. McHattie et al. (1999) observed an internalization of PrP106-126 by microglia, neurons and astrocytes [13]. They further showed that this uptake is independent of PrP^C^ expression, indicating that microglial cells phagocytose prion peptides per se and that this uptake is at least not mediated through the PrP^C^ protein.

Our results support the assumption that microglial cells are involved in prion clearance by phagocytosis of the prion protein. We demonstrated that microglial cells phagocytosed the prion peptide PrP106-126 A117V in its fibrillated form in a time-dependent manner. We assume this uptake to be beneficial, as we could not detect any release of either cytokines or NO in cultures exposed to 10 µM PrP106-126 A117V. These findings contrast with other studies demonstrating microglial activation following 80 µM PrP exposure, which indicates that higher concentrations of PrP106-126 A117V might be necessary to activate an inflammatory response in microglia [34], [35].

Prion peptides have a toxic effect on primary neuronal cells. This toxic effect is prevented by the co-incubation of prion peptides with naturally occurring autoantibodies against the prion protein (nAbs-PrP) [3]. These results indicate a beneficial effect of nAbs-PrP in terms of prion toxicity and raise hope for a possible therapeutic strategy. Given the beneficial effect of IVIg in clinical trials of Alzheimer’s disease patients [36], naturally occurring antibodies have been considered a useful therapeutic agent for neurodegenerative diseases [6], [37]. In this paper, we demonstrated that nAbs-PrP enhanced the uptake of prion peptides in primary microglial cells. This effect was specific for nAbs-PrP, as we did not see the same effect with ft-PrP. The increased uptake of prion peptides did not result in an inflammatory response of microglial cells. However, the uptake of PrP106-126 A117V seemed to result in a drop in mitochondrial activity as assessed by MTT assay. nAbs-PrP and ft-PrP applied alone did not elicit the same effect. However, the use of MTT assay in combination with amyloidogenic peptides has limitations because amyloidogenic peptides bear the ability to enhance the exocytosis of the reduced tetrazolium dye in cells [22]. We therefore applied a second method to further examine the viability of microglial cells following prion and antibody exposure. Interestingly, by staining microglial cells with fluorescein diacetate/propidium iodide, we detected an increase in cell count after exposing cells to PrP106-126 A117V with or without antibodies. This result implies cell proliferation rather than cell death. These findings are in line with previous studies demonstrating an induced microglial proliferation following PrP106-126 exposure [38].

We could not detect any effect on the viability of neuronal cells following exposure to supernatants from microglia exposed to PrP106-126 A117V and nAbs-PrP. Furthermore, the application of nAbs-PrP and ft-PrP alone did not induce any inflammatory response or toxic effects on microglia or neurons. This finding reveals an important feature when considering IVIg and/or nAbs-PrP as possible treatment options. Because our data represent an *in vitro* model only, it further needs to be verified whether nAbs-PrP also influence microglia *in vivo*. There is evidence from immunotherapy studies that peripherally applied antibodies are able to pass the intact blood–brain barrier [39], [40], [41]. We have shown that ^111^In-labelled naturally occurring autoantibodies against Aβ cross the blood–brain barrier in the APP23 transgenic mouse model of Alzheimer’s disease [42]. From these data, it can be concluded that a certain amount of peripherally administered nAbs-Aβ are able to cross the blood–brain barrier. Therefore, we hypothesize that nAbs-PrP can as well because these are quite similar to nAbs-Aβ. In our experiments, we used microglia of mouse origin. Experiments by Fabrizi et al. (2001) revealed a similar behavior of human microglial cells following PrP exposure compared to the murine microglial cells used by Brown et al. (1996) [34], [38]. We therefore hypothesize that the comparability of cells from different species also applies for the application of nAbs-PrP. However, further experiments are necessary to address the differences between microglial cells from different origin.

The effects of nAbs-Aβ on the phagocytosis of Aβ and the viability of microglial cells have been analyzed in a recent communication by our group [43]. In contrast to our experiments with nAbs-PrP, Gold et al. (2013) found a profound inflammatory response following the *in vitro* treatment of microglia with co-administration of oligomerized Aβ and nAbs-Aβ. With respect to the inflammatory reactions observed in response to challenge with different types of oligomers, microglial cells may react in a variety of ways [44]. The impact of nAbs-PrP or nAbs-Aβ on those processes and the underlying signaling pathways are not yet fully understood. However, in treatment with nAbs-PrP or nAbs-Aβ alone, we did not observe any change in cytokine production, whereas Gold et al. (2013) detected a slight increase in cytokine concentrations. This discrepancy might have been caused by the different types of antibodies used (i.e., nAbs-PrP or nAbs-Aβ). Because IVIg itself induces an inflammatory response in microglial cells [45], it seems reasonable that the different antibody preparations isolated from IVIg might induce a variety of responses in microglia. Moreover, the *in vivo* experiments by Gold et al. (2013) did not demonstrate any inflammatory reaction following the administration of nAbs-Aβ in Tg2576 mice. These results indicate the need for additional *in vivo* studies to further evaluate the action of nAbs-PrP on inflammatory reactions *in vivo*.

Our results suggest that nAbs-PrP contribute to the clearance function of microglial cells without leading to an inflammatory response, thus triggering neuronal loss. In our study, we applied 10 µM PrP106-126 A117V, compared to 80 µM PrP in other studies. Our findings indicate that microglia might only be deleteriously activated by excessive prion accumulation, but low concentrations might not lead to an activation that causes cell damage. It may be concluded that nAbs-PrP are important for prion clearance and have no detrimental side effects. However, our results were obtained using PrP106-126 A117V peptides only. It still needs to be verified if the same results can be achieved by using full-length PrP. Preliminary experiments revealed highly specific binding of nAbs-PrP to human recombinant PrP23-231 (data not shown). Wei et al. (2012) further demonstrated nAbs-PrP to successfully immunoprecipitate PrP (A117V) from PrP (A117V) transgenic mice [3]. These results provide evidence for a similar mode of interaction of nAbs-PrP with other PrP peptides. Another limitation, however, is that our studies were performed with single cell culture systems only. Further studies with co-culture systems are needed to characterize these effects in an interactive environment.

To date, not much is known about the underlying mechanisms of prion uptake in microglia. Filamentous actin is required for phagocytosis in general. Cytochalasin D is an inhibitor of actin depolymerization and inhibits scavenger-, complement- and Fcγ receptor-mediated phagocytosis [46], [47]. We demonstrate here that the uptake of prion peptide was almost completely prevented when incubating the cells with cytochalasin D, indicating one of these mechanisms underlies prion peptide uptake. The scavenger receptors are essential for the uptake of Aβ in its fibrillated state [27], [28]. Therefore, we further investigated the role of scavenger receptors and found that they were also involved in the uptake of PrP106-126 A117V fibrils. However, uptake of co-preparations of PrP106-126 A117V and nAbs-PrP or ft-PrP did not seem to be mediated through this pathway, as those were not affected as much by fucoidan as PrP106-126 A117V alone was. In co-preparations of PrP106-126 A117V with nAbs-PrP, wortmannin effectively inhibited its microglial uptake. Wortmannin is a specific PI-3K inhibitor that prevents pseudopod extension of the cells during phagocytic processes. Especially for Fc receptor-mediated phagocytosis, pseudopod extension (and PI-3K activity) is important for the engulfment of particles [48]. In the present study we used microglial cells of murine origin and human IgG. Human Fcγ receptor and murine Fcγ receptor share 65–75% identity in their extracellular domains, and human Fcγ receptor can bind murine IgG [49]. So far, it is not known whether the murine Fcγ receptor can bind to human IgG. Recently, Smith et al. (2012) introduced a mouse model in which murine Fcγ receptors have been replaced by human Fcγ receptors [50]. It might be worth testing nAbs-PrP on microglia from this mouse to exclude the possible impact of species differences on the interaction of immunoglobulin and Fc receptor. However, PI-3K activity is also involved in complement receptor-mediated phagocytosis, even if this process occurs rather passively with the appearance of only small pseudopodia [51]. Comparison of the phagocytic characteristics after fucoidan treatment alone, in contrast to fucoidan/wortmannin co-treatment, revealed that additional wortmannin mainly affected the uptake of PrP106-126 A117V co-administered with nAbs-PrP. This finding was supported by further experiments that investigated the effects of cytochalasin D, fucoidan and wortmannin on the uptake of nAbs-PrP and ft-PrP. We found that only wortmannin pre-treatment of microglial cells resulted in a markedly reduced uptake of nAbs-PrP and ft-PrP. These findings indicate that the uptake of nAbs-PrP or ft-PrP alone is mainly achieved through PI-3K-mediated phagocytosis. Because our data show that the uptake of PrP106-126 A117V fibrils is at least partly scavenger receptor-mediated but phagocytosis of nAbs-PrP and ft-PrP occurs mainly through PI-3K mediated pathways, we conclude that there are at least two different mechanisms involved in the uptake of PrP106-126 A117V and nAbs-PrP. However, the mechanism that is mainly responsible for prion uptake and whether nAbs-PrP merely support the uptake or give rise to a completely new mechanism of prion uptake needs to be further elucidated.

In summary, microglial cells are activated during prion disease and thus contribute to neurodegeneration. In contrast, these cells function in the clearance of prion proteins, and insufficient prion clearance is a possible cause of severe prion accumulation [52]. In infected mice, prion accumulation occurs in large plaques. In our experiments, comparably lower concentrations were applied. One possibility is that microglial clearance of prion proteins is only functional under low concentrations and/or lower aggregates of prion protein. The presence of larger aggregates therefore disrupts this ability. From our experiments, it seems reasonable to administer nAbs-PrP to help microglial cells to clear the extracellular space at the very beginning of prion accumulation. Our data also show that this clearance mechanism takes place without any inflammatory response and by avoiding neuronal cell death. Given the beneficial effects and few adverse side effects of IVIg administration in clinical studies, nAbs-PrP might be a target for therapeutic aspects of prion diseases. However, we must emphasize that our data are from an *in vitro* model. Therefore, further work using animal models is required to determine whether these results also apply to the *in vivo* situation.
